# Editorial: Smart devices for personalized nutrition and healthier lifestyle behavior change

**DOI:** 10.3389/fnut.2025.1604314

**Published:** 2025-04-29

**Authors:** M. Fátima Domingues, Kosmas Dimitropoulos, Kathryn Hart, Sofia Balula Dias

**Affiliations:** ^1^Department of Biomedical Engineering and Biotechnology and Healthcare Engineering Innovation Group (HEIG), Khalifa University of Science and Technology, Abu Dhabi, United Arab Emirates; ^2^The Visual Computing Lab, Information Technologies Institute, Centre for Research and Technology Hellas, Thessaloniki, Greece; ^3^School of Biosciences, Faculty of Health and Medical Sciences, University of Surrey, Guildford, United Kingdom; ^4^Center of Interdisciplinary Study of Human Perfomance (CIPER), Faculdade de Motricidade Humana, Universidade de Lisboa, Lisbon, Portugal

**Keywords:** personalized nutrition, healthy eating, mobile health, smart technologies, dietary assessment, personalized recommendations, disease management, artificial intelligence

## Introduction

Smart devices have emerged as transformative tools at the intersection of digital health, nutrition science, and behavioral change, offering innovative solutions to address pressing public health challenges. By leveraging advancements in artificial intelligence (AI), machine learning, and mobile health (mHealth) technologies, these devices enable personalized interventions tailored to individual needs and preferences, ultimately driving significant improvements in health outcomes (1–3). Recent studies have highlighted the potential of smart technologies to promote healthier eating habits, increase physical activity, and manage chronic health conditions through personalized interventions. AI-driven applications, such as the PROTEIN Advisor and similar tools, are capable of analyzing dietary patterns and delivering expert-validated meal recommendations tailored to user preferences (4–6). These innovations have demonstrated their utility in addressing conditions like diabetes and obesity through tailored nutritional and physical activity plans. Furthermore, recent advances in Large Language Models (LLMs) have led to the development of AI-powered advisors capable of providing personalized nutritional recommendations (7). Finally, mHealth platforms have proven effective in promoting sustained behavior change, particularly among underserved populations and aging demographics, by combining user-centered design with data-driven strategies (2, 8).

Healthcare is shifting toward prevention and patient empowerment, with smart technologies in personalized nutrition enabling healthier behaviors, reducing chronic disease risks, and addressing diverse needs. Advancing this field will rely on interdisciplinary collaboration between nutrition, digital health, and behavioral psychology to create effective, global health solutions. Aligned with the theme *Smart Devices for Personalized Nutrition and Healthier Lifestyle Behavior Change*, this Editorial emphasizes six contributions from the *Frontiers in Nutrition* journal that showcase the potential of these advancements. These studies span interdisciplinary areas such as Nutrition and Food Science Technology, Public Health and Nutrition, and Digital Public Health, showcasing the diversity and depth of this research. Notable innovations include mHealth interventions designed to enhance health literacy and foster positive lifestyle changes, digital platforms tailored for managing chronic conditions, and AI-driven tools for dietary assessment and nutritional risk prediction. Collectively, these efforts demonstrate the value of integrating clinical nutrition practices with emerging technologies and behavioral theories to create holistic health solutions.

Moreover, the contributions emphasize the practical applications of these technologies in real-world scenarios, such as empowering underserved populations to access preventive care, promoting sustained exercise for chronic disease management, and developing dietary quality indices for specific demographics like pregnant women. These advances reflect a broader shift away from traditional approaches tailored at the group or population level toward personalized, data-driven health interventions, where smart devices act as pivotal facilitators of behavior change and self-management. They also underscore the critical role of interdisciplinary collaboration, combining expertise from nutrition, technology, and public health, to effectively address complex health challenges.

The Research Topic shows the transformative role of user-centered design, data-driven strategies, and smart devices in modern health ecosystems. As this field continues to evolve, these contributions set a solid foundation for future research and applications, driving advancements in personalized nutrition, public health strategies, and sustainable behavior modification. Through this body of work, a compelling vision emerges for leveraging technology to achieve impactful and accessible health improvements globally.

As Guest Editors for the *Frontiers in Nutrition* journal, we are proud to present this Research Topic, which has undergone a rigorous peer review process and reflects the insights of high-caliber international reviewers and guest editors. Together, these papers make significant contributions to the existing body of knowledge and open new pathways for innovation and progress in nutrition science, public health, and digital health solutions.

## Contributions

**Figure 1:**
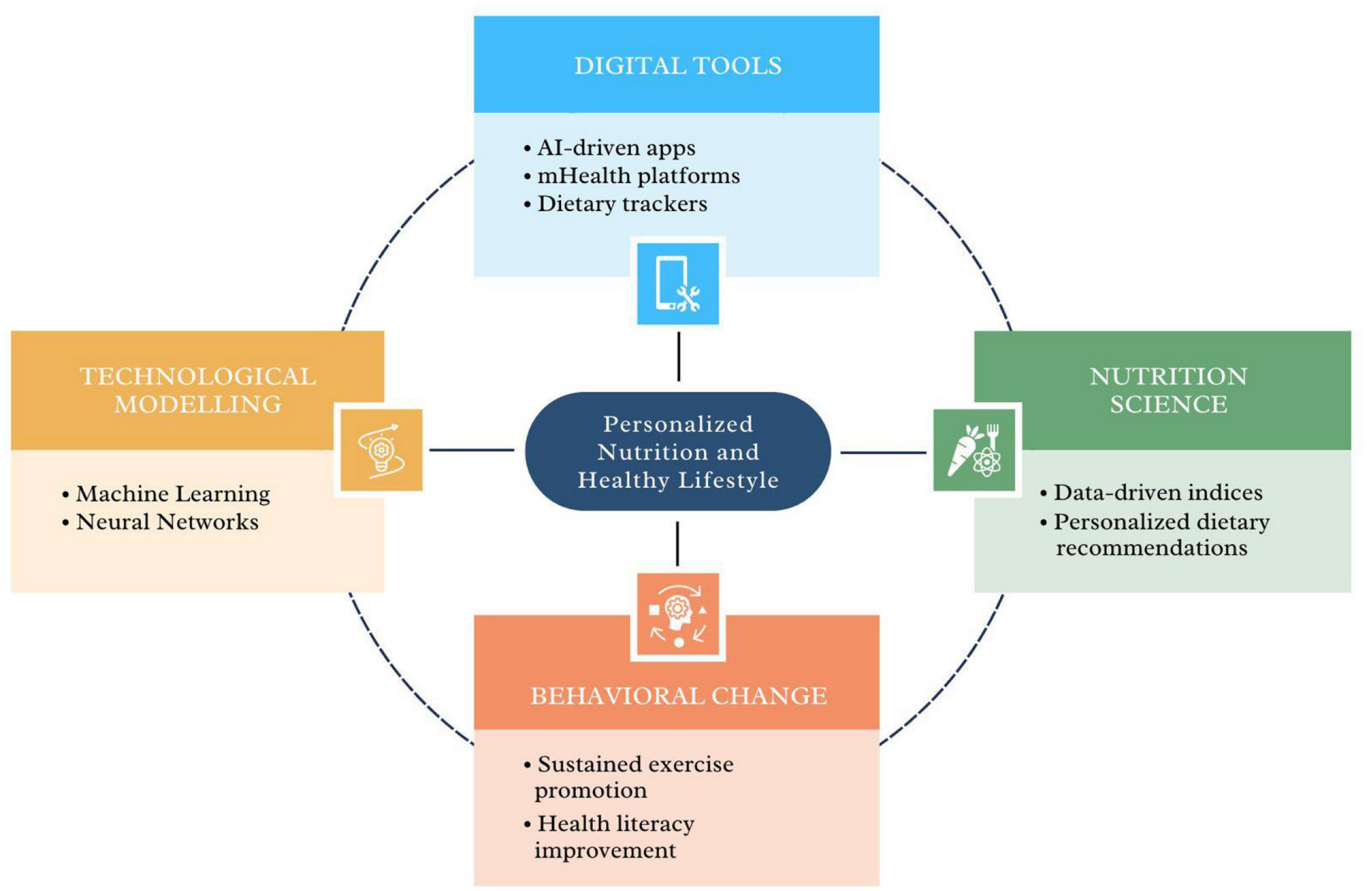
An overview of personalized health domains, showcasing digital tools like AI-driven apps, mHealth platforms, and dietary trackers; progress in nutrition science with data-driven indices and personalized dietary recommendations; behavioral change strategies such as promoting sustained exercise and enhancing health literacy; and computation modeling, including machine learning and neural networks models.

This Research Topic showcases innovative research and applications across digital health, nutrition, and behavioral change. It includes efforts to enhance health awareness, encourage sustained exercise for chronic disease management, and to develop tools for dietary self-tracking and pregnancy-specific nutrition evaluation. Additionally, advancements in AI models for nutritional risk prediction and neural networks for recipe analysis underscore the integration of technology into personalized health solutions. These studies collectively highlight the potential of combining digital tools and behavioral approaches to improve health outcomes (see Figure 1).

Rani et al. evaluate the mDiabetes intervention, a program aimed at improving diabetes awareness and encouraging healthier lifestyle habits among rural populations in India. Using a quasi-experimental design, the study involved over 100,000 participants who received diabetes prevention messages via voice calls in their local language over 6 months. Community health education sessions and informational leaflets complemented the intervention. Results showed significant improvements in diabetes awareness, with rates increasing from 82.75% at baseline to 99.63% at follow-up. Additionally, beliefs in diabetes preventability and the role of lifestyle in diabetes management also saw notable increases. The study highlights the effectiveness of mHealth tools in addressing public health challenges in underserved areas.

Alves et al. introduces a digital health solution aimed at encouraging sustained exercise participation among individuals with Parkinson's Disease (PwPD). The solution includes a web platform and a mobile app with a conversational agent, designed using social cognitive theory principles to foster behavior change. A mixed-methods study involving physiotherapists and PwPD assessed the system's usability and acceptability. Results showed high usability scores and positive feedback, highlighting the potential of the MoveONParkinson app to enhance self-management, user engagement, and overall quality of life for PwPD.

Kavanagh et al. evaluates the validity of the clinical Portfolio Diet Score used in the PortfolioDiet.app, a digital tool for dietary self-tracking among adults with hyperlipidemia. Through a simulation model and secondary analysis of data from a randomized controlled trial, the study assesses how accurately the diet score reflects adherence to the Portfolio Diet, a cholesterol-lowering dietary pattern. Results indicate that the Portfolio Diet Score is a reliable measure for monitoring dietary compliance, making the app a useful resource for self-management and clinical research in hyperlipidemia. Overall, the study stresses the potential of such tools to support personalized dietary interventions.

Faessen et al. introduces a diet quality index tailored for pregnant women. The Dutch Health Diet for pregnant women (DHD)-P index consists of 22 components aligned with the 2021 Dutch dietary guidelines for pregnancy. It was evaluated using a Food Frequency Questionnaire (FFQ) and 24-h dietary recalls among pregnant women at 12 and 24 weeks of gestation. The study found moderate to good correlations between the FFQ and recall data, indicating the index's reliability. The DHD-P aims to assess diet quality and provide feedback to promote healthier food choices during pregnancy. Further research is suggested to validate its sensitivity to dietary changes.

Wang et al. discuss the development of a machine learning model that predicts nutritional risk by analyzing facial features. Using advanced image recognition techniques, the model identifies physical indicators associated with nutritional deficiencies or risks. The approach leverages large datasets to train the algorithm for accurate detection and prediction. The study shows the potential of this non-invasive method for early nutritional risk assessment, which could be particularly valuable in clinical settings and underserved populations. Further validation is suggested to improve its reliability and applicability.

Finally, Li et al. present a neural network model designed specifically to analyze and evaluate the nutritional content of recipes. The model utilizes advanced techniques, including image recognition and semantic analysis, to accurately identify and quantify nutrients in food. By incorporating multiple datasets, such as Recipe1M+ and Food2K, the model achieves both precision and adaptability. The study aims to empower individuals to make informed dietary decisions by providing detailed nutritional insights derived from food images and recipes. This groundbreaking work successfully bridges the gap between technology and personalized nutrition.

## Summary

This Research Topic highlights diverse yet significant advancements in digital health, nutrition, and behavioral change, demonstrating the potential of innovative tools and personalized solutions to improve health outcomes. It showcases a wide range of applications, including mHealth interventions for lifestyle changes, digital platforms for chronic disease management, and diet-tracking tools for specific populations such as pregnant women and adults with hyperlipidemia. Additionally, AI-driven models for nutritional risk prediction and recipe analysis emphasize the integration of technology in personalized health management. These studies collectively underline the importance of combining digital tools, behavioral theories, and user-centered approaches to address public health challenges and promote healthier living.
